# Predicting light-induced stomatal movements based on the redox state of plastoquinone: theory and validation

**DOI:** 10.1007/s11120-019-00632-x

**Published:** 2019-03-19

**Authors:** Johannes Kromdijk, Katarzyna Głowacka, Stephen P. Long

**Affiliations:** 1 Carl R. Woese Institute for Genomic Biology, University of Illinois at Urbana-Champaign, 1206 West Gregory Drive, Urbana, IL 61801 USA; 2 Department of Plant Sciences, University of Cambridge, Downing Site, Cambridge, CB23EA UK; 3 Institute of Plant Genetics, Polish Academy of Sciences, 60-479 Poznan, Poland; 4 Lancaster Environment Centre, University of Lancaster, Bailrigg, LA1 1YX UK; 5 Present Address: Department of Biochemistry, University of Nebraska-Lincoln, N246 Beadle Center, 1901 Vine Street, Lincoln, NE USA

**Keywords:** Stomatal conductance model, Light response, Plastoquinone, Chlorophyll fluorescence, Gas exchange, Crop models

## Abstract

Prediction of stomatal conductance is a key element to relate and scale up leaf-level gas exchange processes to canopy, ecosystem and land surface models. The empirical models that are typically employed for this purpose are simple and elegant formulations which relate stomatal conductance on a leaf area basis to the net rate of CO_2_ assimilation, humidity and CO_2_ concentration. Although light intensity is not directly modelled as a stomatal opening cue, it is well-known that stomata respond strongly to light. One response mode depends specifically on the blue-light part of the light spectrum, whereas the quantitative or ‘red’ light response is less spectrally defined and relies more on the quantity of incident light. Here, we present a modification of an empirical stomatal conductance model which explicitly accounts for the stomatal red-light response, based on a mesophyll-derived signal putatively initiated by the chloroplastic plastoquinone redox state. The modified model showed similar prediction accuracy compared to models using a relationship between stomatal conductance and net assimilation rate. However, fitted parameter values with the modified model varied much less across different measurement conditions, lessening the need for frequent re-parameterization to different conditions required of the current model. We also present a simple and easy to parameterize extension to the widely used Farquhar–Von Caemmerer–Berry photosynthesis model to facilitate coupling with the modified stomatal conductance model, which should enable use of the new stomatal conductance model to simulate ecosystem water vapour exchange in terrestrial biosphere models.

**Electronic supplementary material:**

The online version of this article (10.1007/s11120-019-00632-x) contains supplementary material, which is available to authorized users.

## Introduction

Terrestrial plants need to take up water from the surrounding environment, retain or transfer water internally, as well as acquire carbon dioxide from the surrounding air to drive photosynthetic carbon assimilation. This trade-off between optimizing carbon uptake via atmospheric diffusion versus minimizing water loss to the atmosphere drove the evolution of highly specialized, controllable stomatal pores in the epidermis of plant leaves (Chater et al. 2017). Stomatal pores are flanked by a pair of guard cells, the only photosynthetic cells of the epidermis, in which turgor changes regulate the pore’s aperture in response to a variety of cues (Kollist et al. 2014), such as leaf (and plant) water status (Mott and Parkhurst 1991; Whitehead 1998), carbon dioxide concentration (Engineer et al. 2016) and light (Assman and Shimazaki 1999; Kaiser and Kappen 1997). The importance of the control of stomatal aperture for plant fitness is clear. Stomatal conductance to water vapour strongly determines transpiratory water loss at leaf-level (Pearcy et al. 1989) and this relationship can be scaled to canopy transpiration, when accounting for leaf area, canopy conductance and degree of coupling between the canopy and atmosphere (e.g. Mielke et al. 1999). In doing so, it can be shown that stomatal movements significantly influence ecosystem water (and energy) exchange (Wehr et al. 2017). In fact, recent estimates show that transpiratory water loss through stomata accounts for 43–75% of global terrestrial evapotranspiration (Wei et al. 2017). This importance of stomatal conductance as a control factor for gaseous fluxes across spatial scales emphasizes the need for robust stomatal conductance models to accurately simulate changes in response to—and interactions with—the surrounding environment in current and future climate scenarios.

A wide variety of models for stomatal conductance exist, ranging from very detailed to more simplified descriptions (for reviews, see Buckley 2017; Damour et al. 2010). The majority of stomatal conductance models cover only steady state responses, although significant progress is being made to capture dynamic behaviour of stomatal conductance (Bellasio et al. 2017; Vialet-Chabrand et al. 2017; Wang et al. 2017). Despite these advances, the empirical Ball–Woodrow–Berry (BWB) model (Ball et al. 1986) is still the most widely used prediction tool for stomatal conductance in models extending across spatial scales. The BWB model is a very simple, elegant formulation, which relates (steady state) stomatal conductance to the humidity and CO_2_ concentration of air surrounding the leaf, and the prevailing rate of photosynthesis, using slope (*g*_1_) and intercept (*g*_0_) parameters. The simplicity of the BWB model facilitates easy coupling to the Farquhar–Von Caemmerer–Berry (FvCB) photosynthesis model (Farquhar et al. 1980), which has been convenient for use in leaf and canopy gas exchange models, as well as ecosystem and land surface models for climate simulation (Bonan et al. 2014).

The original BWB model considers humidity as a percentage of saturated vapour pressure, but this has been altered in several modified versions to a parameter based on vapour pressure deficit (e.g. Dougherty et al. 1994; Leuning 1995; Medlyn et al. 2011). Additional dependencies on soil moisture, plant water status and abscisic acid concentration (Tenhunen et al. 1990; Wang and Leuning 1998; Gutschick and Simonneau 2002) can also be added. The effects of CO_2_ on stomatal movements are directly accounted for via multiplication with the inverse of ambient CO_2_ concentration, as well as via an implicit feedback through multiplication with net CO_2_ assimilation rate (*A*_*n*_), which is itself responsive to CO_2_ concentration. The influence of light on stomatal movements is not explicitly accounted for in the BWB model, but is implicitly assumed to be equal to the effects of light on *A*_*n*_, thus assuming a direct link between photosynthesis and light-induced stomatal movements. Whereas this assumption is a convenient approximation, it is not consistent with current understanding of light-induced stomatal movements. Instead, light affects stomatal movements in at least two separate ways. Firstly, illumination with (low intensity) blue light activates phototropins, blue-light photoreceptors, which in turn activate a signal transduction chain leading to stomatal opening (Inoue and Kinoshita 2017). These blue-light effects can be most clearly observed in the background of red-light illumination, which also stimulates stomatal opening. However, whereas several signalling components of the blue-light response of stomatal opening have been elucidated, the ‘quantitative’ or ‘red light’ response of stomatal conductance is less well understood. Some evidence suggests that phytochromes A and B, red:far-red light photoreceptors, might be involved (Wang et al. 2010) as well as a specific set of MYB transcription factors (*At*MYB60 and *At*MYB61, Liang et al. 2005). Additionally, whereas the blue-light response appears entirely located in the guard cells, the red-light response seems to depend on a mesophyll-derived signal (Mott et al. 2008; Lawson et al. 2014). This signal was long assumed to be directly related to photosynthesis, but stomatal conductance in plants with transgenically decreased photosynthetic capacity was not decreased proportionally (e.g. Von Caemmerer et al. 2004; Baroli et al. 2008; Lawson et al. 2008), providing evidence that the mesophyll signal does not scale directly with photosynthetic rates. Additionally, responses to red light cannot simply be explained by concomitant effects on intercellular CO_2_ concentration (*C*_i_), since stomata still respond to red light when *C*_i_ is kept constant (Messinger et al. 2006). Busch (2014) suggested that instead of a photosynthesis-derived signal, the redox state of the chloroplastic plastoquinone (PQ) pool might be signalled to the stomatal guard cells. Consistent with this hypothesis, we recently observed tightly and linearly coordinated changes in the redox state of quinone A, estimated by fluorescence parameter *1* − *q*_L_ (Kramer et al. 2004) and stomatal conductance in tobacco with modified levels of photosystem II subunit S (PsbS) (Głowacka et al. 2018), which is a strong determinant of the amplitude of non-photochemical quenching and therefore also affects the redox state of the chloroplastic electron transport chain.

In the current manuscript we present a modified stomatal conductance model, which explicitly accounts for these observed responses. The parameterization of the resulting model is demonstrated to be less sensitive to measurement conditions compared to the BWB models which simulate stomatal conductance as a function of net assimilation rate. We also show that a simple extension to the FvCB photosynthesis model can be used to predict *1* − *q*_L_ from combined gas exchange and chlorophyll fluorescence measurements, which facilitates coupling to the modified stomatal conductance model.

## Materials and methods

### Modified stomatal conductance model

The BWB model (Ball et al. 1986) calculates stomatal conductance to water vapour from a linear product of net assimilation rate *A*_*n*_, relative humidity *h*_s_ and the inverse of CO_2_ concentration surrounding the leaf (*C*_a_). Here we use the recent version by Medlyn et al. (2011) as a starting point, where the inverse square-root of atmospheric vapour pressure deficit (VPD_A_) is used instead of *h*_s_ to capture effects of humidity on *g*_s_. The resulting term is scaled empirically to measured stomatal conductance, using a slope parameter *g*_1_ and intercept parameter *g*_0_, such as shown in Eq. (1).

1 $${g_{\text{s}}}={g_0}+1.6 \times \left( {1+\frac{{{g_1}}}{{\sqrt {{\text{VP}}{{\text{D}}_{\text{A}}}} }}} \right) \times \frac{{{A_n}}}{{{C_{\text{a}}}}}$$

Recent data (Głowacka et al. 2018) suggest that the stomatal ‘quantitative’ or ‘red’ light response may be initiated by a PQ redox signal, which we approximate by *1* − *q*_L_ i.e. the redox state of the quinone bound to the *Q*_A_ site at photosystem II (PSII). We therefore replaced *A*_*n*_ in Eq. (1) with (*1* − *q*_L_) (Eq. 2). Note that the empirical constants in Eq. (1) are used similarly to Eq. (2) but have been renamed, to facilitate easy comparison between parameter estimation based on the Medlyn model and the modified model.

2 $${g_{\text{s}}}={g_{0,{\text{new}}}}+1.6 \times \left( {1+\frac{{{g_{1,{\text{new}}}}}}{{\sqrt {{\text{VP}}{{\text{D}}_{\text{A}}}} }}} \right) \times \frac{{1~ - ~{q_{\text{L}}}}}{{{C_{\text{a}}}}}$$

### Extension of the FvCB photosynthesis model to simulate *q*_L_

The biochemical model for leaf photosynthesis by Farquhar et al. (1980; abbreviated as FvCB model) is widely used in conjunction with stomatal models such as Eq. (1). Coupling between the FvCB model and the new stomatal conductance model in Eq. (2) would require simulation of *q*_L_. Therefore, we present a simple extension of the FvCB model to allow simulation of *q*_L_. The FvCB model has a switch-point structure and simulates net assimilation rate as the minimum of three limiting factors: RuBP carboxylation-limited rate (*A*_c_), RuBP regeneration-limited rate (*A*_j_) and triose phosphate utilization limited rate (*A*_TPU_).

3a $${A_{\text{c}}}=\frac{{{V_{{{\text{cmax}}}}} \times ({C_{\text{c}}} - {\Gamma ^*})}}{{{C_{\text{c}}}+{K_{\text{c}}} \times \left( {1+\frac{{{O_{\text{c}}}}}{{{K_{\text{o}}}}}} \right)}} - {R_{\text{d}}}$$

3b $${A_{\text{j}}}=\frac{{J \times ({C_{\text{c}}} - {\Gamma ^*})}}{{4{C_{\text{c}}}+8{\Gamma ^*}}} - {R_{\text{d}}}$$

3c $${A_{{\text{TPU}}}}=3{V_{{\text{TPU}}}} - {R_{\text{d}}}$$

3d $${A_{\text{n}}}=\hbox{min} \left( {{A_{\text{c}}},\;{A_{\text{j}}},\;{A_{{\text{TPU}}}}} \right)$$

here $V_{\text{cmax}}$ is the maximal rate of RuBP carboxylation and *K*_c_ and *K*_o_ are the Michaelis–Menten constants to describe CO_2_ and O_2_ effects on RuBP carboxylation. *C*_c_ represents the chloroplastic CO_2_ concentration, Γ* represents the CO_2_ compensation point in the absence of *R*_d_ and *R*_d_ represents mitochondrial respiration not associated with photorespiration. *V*_TPU_ is the maximal rate of triose phosphate utilization and *O*_*c*_ represents the O_2_ concentration in the chloroplast, which was assumed to equal ambient.

Next, the rate of whole-chain electron transport (*J*; Eq. 4) was modelled as a function of absorbed light intensity (PFD_abs_) using a non-rectangular hyperbola, with initial slope *α*, asymptote *J*_max_ and shape factor *θ*.

4 $$J=\frac{{\alpha \times {f_{{\text{PSII}}}} \times {\text{PF}}{{\text{D}}_{{\text{abs}}}}+{J_{\text{max} }} - \sqrt {{{(\alpha \times {f_{{\text{PSII}}}} \times {\text{PF}}{{\text{D}}_{{\text{abs}}}})}^2} - 4 \times \theta \times \alpha \times {f_{{\text{PSII}}}} \times {\text{PF}}{{\text{D}}_{{\text{abs}}}}+{J_{\text{max} }}} }}{{2 \times \theta }}$$

here *f*_PSII_ represents the proportion of absorbed light partitioned to PSII. The level of *J* was used to calculate the operating efficiency of photosystem II (*Φ*_PSII_) at a given light level:

5 $${\Phi _{{\text{PSII}}}}=\frac{J}{{{\text{PF}}{{\text{D}}_{{\text{abs}}}} \times {f_{{\text{PSII}}}}}}$$

To describe the steady state level of non-photochemical quenching (NPQ) as a function of light intensity (PFD), a sigmoidal Hill function was used (Eq. 6a), with basal level NPQ_0_, light intensity at half amplitude (*K*_NPQ_), Hill coefficient (*n*NPQ) and asymptote (NPQ_max_). The level of NPQ at the PFD = 0 limit was assumed to equal zero (Eq. 6b).

6a $${\text{PFD}}>{\text{ }}0 \to {\text{NPQ}}=\frac{{{\text{NP}}{{\text{Q}}_{\text{max} }} - {\text{NP}}{{\text{Q}}_0}}}{{\left[ {{{\left( {\frac{{{K_{{\text{NPQ}}}}}}{{{\text{PF}}{{\text{D}}_{{\text{abs}}}}}}} \right)}^{n{\text{NPQ}}}}+1} \right]}}+~{\text{NP}}{{\text{Q}}_0}$$

6b $${\text{PFD}}=0 \to {\text{NPQ}}=0$$

Maximal fluorescence without dark-adaptation at a given light level (${F^{\prime}_{\text{m}}}$) was calculated using NPQ (from Eqs. 6a, 6b) and dark-adapted maximal fluorescence *F*_*m*_ according to Eq. (7). The corresponding level of *F*′ was computed with Eq. (8), using *Φ*_PSII_ from Eq. (5):

7 $${F^{\prime}_{\text{m}}}=\frac{{{F_{\text{m}}}}}{{{\text{NPQ}}+1}}$$

8 $$F^{\prime}=\frac{{{{F^{\prime}}_{\!\!\text{m}}}}}{{1 - {\Phi _{{\text{PSII}}}}}}$$

To predict minimal fluorescence without dark-adaptation (${F^{\prime}_{\text{o}}}$) as a function of light intensity, we separately considered effects of suppression of fluorescence via *NPQ* and elevation of fluorescence due to photo-inactivated reaction centres. The decrease in ${F^{\prime}_{\text{o}}}$ relative to *F*_o_ as a result of *NPQ* (calculated as ${F^{\prime}_{{\text{oNPQ}}}}$) was estimated from ${F^{\prime}_{\text{m}}}$ and *F*_o_ according to Oxborough and Baker (1997):

9 $${F^{\prime}_{{\text{oNPQ}}}}=\frac{{{F_{\text{o}}}}}{{\frac{{{F_{\text{v}}}}}{{{F_{\text{m}}}}}+\frac{{{F_{\text{o}}}}}{{{{F^{\prime}}_{\!\!\text{m}}}}}}}$$

Using ${F^{\prime}_{{\text{oNPQ}}}}$ from Eq. (9), the effects of *NPQ* on the maximal PSII quantum efficiency in the light (${{{{F^{\prime}_{\text{v}}}}} \mathord{\left/ {\vphantom {{{{F^{\prime}_{\text{v}}}}} {{{F^{\prime}_{\text{m}}}}}}} \right. \kern-0pt} {{{F^{\prime}_{\text{m}}}}}}$) can be predicted:

10 $$\left( {\frac{{{{F^{\prime}_{\text{v}}}}}}{{{{F^{\prime}_{\text{m}}}}}}}\right)_{\text{NPQ}}=1 - \frac{{{{F^{\prime}_{{\text{oNPQ}}}}}}}{{{{F^{\prime}_{\text{m}}}}}}$$

Next, we used an empirical relationship to predict the elevation of minimal fluorescence due to inactivation of reaction centres. Hendrickson et al. (2005) showed that the energy flux approximated by $0.5 \times {\text{PF}}{{\text{D}}_{{\text{abs}}}} \times \frac{{F^{\prime}}}{{{{F^{\prime}}_{\text{m}}}}}$ is a reasonable estimator of the rate constant of photo-inactivation. Therefore, we predicted the relative difference between $\left( {{{{{F^{\prime}_{\text{v}}}}} \mathord{\left/ {\vphantom {{{{F^{\prime}_{\text{v}}}}} {{{F^{\prime}_{\text{m}}}}}}} \right. \kern-0pt} {{{F^{\prime}_{\text{m}}}}}}} \right)_{\text{NPQ}}$ from Eq. (10) and observed ${{{{F^{\prime}_{\text{v}}}}} \mathord{\left/ {\vphantom {{{{F^{\prime}_{\text{v}}}}} {{{F^{\prime}_{\text{m}}}}}}} \right. \kern-0pt} {{{F^{\prime}_{\text{m}}}}}}$ by a linear function of $0.5 \times {\text{PF}}{{\text{D}}_{{\text{abs}}}} \times {{F^{\prime}} \mathord{\left/ {\vphantom {{F^{\prime}} {{{F^{\prime}}_{\text{m}}}}}} \right. \kern-0pt} {{{F^{\prime}}_{\!\!\text{m}}}}}$ according to Eq. (11):

11 $$1 - \frac{{\left( {\frac{{{{F^{\prime}_{\text{v}}}}}}{{{{F^{\prime}_{\text{m}}}}}}} \right)}}{{\left( {\frac{{{{F^{\prime}_{\text{v}}}}}}{{{{F^{\prime}_{\text{m}}}}}}} \right)_{\text{NPQ}}}}=m \times \left( {0.5 \times {\text{PF}}{{\text{D}}_{{\text{abs}}}} \times \frac{{F^{\prime}}}{{{{F^{\prime}_{\text{m}}}}}}} \right)+n$$

The empirical coefficients *m* and *n* were fitted on light response curves of chlorophyll fluorescence parameters. Combining Eq. (11) with simulated fluorescence levels from Eqs. (7) and (8) then allowed calculation of *q*_L_ using the formulation by Kramer et al. (2004):

12 $${q_{\text{L}}}=\frac{{{{F^{\prime}}_{\!\!\text{m}}} - F^{\prime}}}{{{F_{\text{m}}} - {F_{\text{o}}}}} \times \frac{{{{F^{\prime}}_{\!\!\text{o}}}}}{{F^{\prime}}}$$

### Coupling the photosynthesis and stomatal conductance models

Using the equations presented above, *q*_L_ can be calculated, which provides a handle for coupling the photosynthesis model with the modified stomatal conductance model. First of all, the intercellular CO_2_ concentration *C*_i_ is dependent on the CO_2_ concentration in the chloroplast *C*_c_ at a given rate of photosynthesis. The value of *C*_i_ could therefore be modelled based on the photosynthesis model using Fick’s law of diffusion (Eq. 13).

13 $${C_{\text{i}}}={C_{\text{c}}}+\frac{{{A_{\text{n}}}}}{{{g_{\text{m}}} \times P}}$$

here *P* represents atmospheric pressure and *g*_m_ is mesophyll conductance to CO_2_. Additionally, *C*_i_ can be predicted from the CO_2_ concentration surrounding the leaf (*C*_a_), the rate of *A*_*n*_, and the value of stomatal conductance (*g*_s_) from Eqs. (1) or (2).

14 $${C_{\text{i}}}=\frac{{\left[ {\frac{1}{{\frac{{1.6}}{{{g_{\text{s}}}}}+\frac{{1.37}}{{{g_{{\text{bl}}}}}}}} - \frac{{{\text{VP}}{{\text{D}}_{\text{L}}}}}{{2 \times P \times \left( {\frac{1}{{{g_{\text{s}}}}}+\frac{1}{{{g_{{\text{bl}}}}}}} \right)}}} \right] \times {C_{\text{a}}} - {A_n}}}{{\left[ {\frac{1}{{\frac{{1.6}}{{{g_{\text{s}}}}}+\frac{{1.37}}{{{g_{{\text{bl}}}}}}}}+\frac{{{\text{VP}}{{\text{D}}_{\text{L}}}}}{{2 \times P \times \left( {\frac{1}{{{g_{\text{s}}}}}+\frac{1}{{{g_{{\text{bl}}}}}}} \right)}}} \right]}}$$

here *g*_bl_ represents the conductance to H_2_O through the leaf boundary layer, VPD_L_ represents leaf-to-air vapour pressure deficit. Using these two formulations for *C*_i_, the models were coupled by iterative minimization of differences between Eqs. (13) and (14).

### Parameter estimation for the Medlyn model and modified stomatal conductance model

The parameters for the stomatal conductance models were estimated using measurements on tobacco plants. Tobacco seeds (*Nicotiana tabacum*, cv ‘Petite Havana’) were germinated on soilless cultivation medium (LC1 Sunshine mix, Sun Gro Horticulture, Agawam, MA, USA) in a controlled environment walk-in growing chamber (Environmental Growth Chambers, Chagrin Falls, OH, USA) with photoperiod set to 12 h and temperature controlled at 25/23 °C (day/night). Five days after germination seedlings were moved to the greenhouse, transplanted to 9 × 4 potting trays (3600 series, Hummert International, Earth City, MO, USA) and grown until two true leaves had emerged. When two true leaves had emerged, seedlings were transplanted to 3.8 L pots (400C, Hummert International, Earth City, MO, USA) filled with growing medium (LC1 Sunshine mix, Sun Gro Horticulture) supplemented with 10 g granulated fertilizer per pot (Osmocote Plus 15/9/12, The Scotts Company LLC, Marysville, OH, USA). Pots were spaced 30 cm apart on greenhouse tables and watered and positions randomized every 2 days.

Gas exchange measurements were performed on the youngest fully expanded leaf after 2.5 weeks of growth (leaf 5), using an open gas exchange system (LI6400XT, LI-COR, Lincoln, Nebraska, USA) equipped with a 2 cm^2^ leaf chamber fluorometer (LCF6400-40, LI-COR), corrected for diffusive leaks between cuvette and the surrounding atmosphere. Two sets of light response curves of photosynthesis, fluorescence and stomatal conductance were used to parameterize the Medlyn and modified stomatal conductance models (see Fig. S1). Leaves were dark-adapted and clamped in the gas exchange cuvette, with block temperature controlled at 25 °C. After measuring *F*_o_ and *F*_m_ chlorophyll fluorescence levels, light intensity was increased stepwise from 0 to 50, 80, 110, 140, 170, 200, 300, 400, 500, 600, 800, 1000, 1500 and 2000 µmol m^−2^ s^−1^. When steady state was achieved (typically at least 15 min waiting time per step), gas exchange parameters were logged and *F*′ and ${F^{\prime}_{\text{m}}}$ were determined using the multiphase flash routine (Loriaux et al. 2013). Additionally, ${F^{\prime}_{\text{o}}}$ was measured by switching the actinic light off briefly while turning on far-red LEDs (*λ*_max_ = 740 nm) to rapidly re-oxidize quinone A. The chlorophyll fluorescence levels at each light intensity were used to compute *q*_L_ according to Eq. (12). For the first set of light response curves, CO_2_ concentration inside the cuvette was controlled at 380 µmol mol^−1^ and the light intensities were achieved solely with red light emitting diodes (*λ*_max_ = 630 nm). This set has been previously published in Głowacka et al. (2018). For set 2, CO_2_ concentration in the reference air was controlled at 1000 µmol mol^−1^ and light intensities were a sum of 90% red and 10% blue (*λ*_max_ = 470 nm) on a photon flux basis. The curves were performed on *n* = 6 biological replicates for set 1 and *n* = 7 for set 2. These measurements resulted in two sets of *g*_s_, *A*_*n*_ and 1 − *q*_L_, which were used to estimate parameters *g*_0_ and *g*_1_ in Eq. (1) as well as *g*_0,new_ and *g*_1,new_ in Eq. (2) via linear regression.

### Parameter estimation for the photosynthesis model

Parameter estimation of the photosynthesis model required measuring the capacity for leaf photosynthetic biochemistry (see Fig. S1). For this purpose, CO_2_ response curves of photosynthesis were performed on the youngest fully expanded leaf (*n* = 6 biological replicates). Leaves were clamped in the gas exchange cuvette with light intensity set to 2000 µmol m^−2^ s^−1^ (10% blue). CO_2_ concentration in the airstream was controlled to 400 µmol mol^−1^, and block temperature set to 25 °C. After steady state had been achieved, CO_2_ was varied from 400 to 300, 200, 100, 75, 400, 400, 500, 600, 700, 800, 1200, 1600 and 1900 µmol mol^−1^. At each CO_2_ concentration, gas exchange values were logged, when the coefficient of variation in net leaf CO_2_ uptake rate (*A*_*n*_) and intercellular CO_2_ concentration (*C*_i_) averaged over 10 s became less than 1% (minimum wait time 1 min, maximum wait time 4 min). $V_{\text{cmax}}$ and *V*_TPU_ were obtained by fitting the photosynthesis model according to Sharkey et al. (2007) and temperature corrections within. Mesophyll conductance (*g*_m_) was not co-fitted but a value of 0.60 mol m^−2^ s^−1^ bar^−1^ at 25 °C was derived separately on a parallel set of tobacco plants, using carbon isotope discrimination measurements in parallel with gas exchange from cryogenic trapping and isotope ratio mass spectrometry as described in Kromdijk et al. (2010) and model equations outlined in Evans and Von Caemmerer (2013). *R*_d_ was estimated as the *y* intercept from the linear regression of *A*_*n*_ versus *J* at low light (Yin et al. 2009), where *J* was obtained from the light response curves as described above. To convert incident to absorbed photon flux in both sets of curves, light absorptance was measured on the same leaf position where gas exchange analysis had also been performed, using an integrating sphere (LI1800, LI-COR) connected to a spectrometer (USB-2000, Ocean Optics Inc, Dunedin, Florida, USA). Incident photon flux was converted to absorbed photon flux (PFD_abs_) using the measured absorptance at the actinic wavelengths used.

### Using the coupled model to predict field observations of *A*_*n*_ and *g*_s_

Survey-style measurements on field-grown tobacco were performed on a bright, hot day (July 21, 2015) at the University of Illinois farm in Urbana (40.11°N, 88.21°W). Early morning measurements had to be delayed until all morning dew of the leaves had evaporated, which occurred around 08:00. Thus, measurements were started at 08:15 and repeated every 90 min until 20:15, just prior to sunset. At each time-point, ambient light intensity was first measured using the external PAR-sensor of the LI6400XT. Subsequently, light intensity in the cuvette was set to equal the ambient intensity (using 90% red and 10% blue), block temperature was set to measured air temperature and CO_2_ concentration in the airstream was set to 400 µmol mol^−1^. Leaves were clamped in the cuvette and gas exchange values were logged as soon as stomatal conductance reached steady rates for 10 s (based on visual assessment of the strip-charts), which happened typically after 1.5–2 min. Parameter estimation for the photosynthesis model was performed using additional CO_2_ and light response curves measured on the field-grown plants. The coupled model was used to predict stomatal conductance based on the parameter estimates for *g*_0_, *g*_1_, *g*_0,new_ and *g*_1,new_ from the plants grown under controlled conditions, as well as using re-calibrated parameter values from a best fit with observations.

### Implementation and model fitting

The equations were implemented in Matlab (Version 8.1.0.604, R2013a, The Mathworks Inc. Natick, MA, USA). Parameter estimation of the stomatal conductance and photosynthesis models was performed using constrained nonlinear minimization (‘fmincon’ algorithm with global search) of least squares differences. Linear regressions were performed with SigmaPlot (Version 14.0, SYSTAT Software Inc., San Jose, California, USA). Re-calibration of the stomatal conductance model under field conditions was performed by minimizing residuals using a grid-search for *g*_0_, *g*_1_, *g*_0,new_ or *g*_1,new_.

## Results

### Stomatal conductance model

**Fig. 1:**
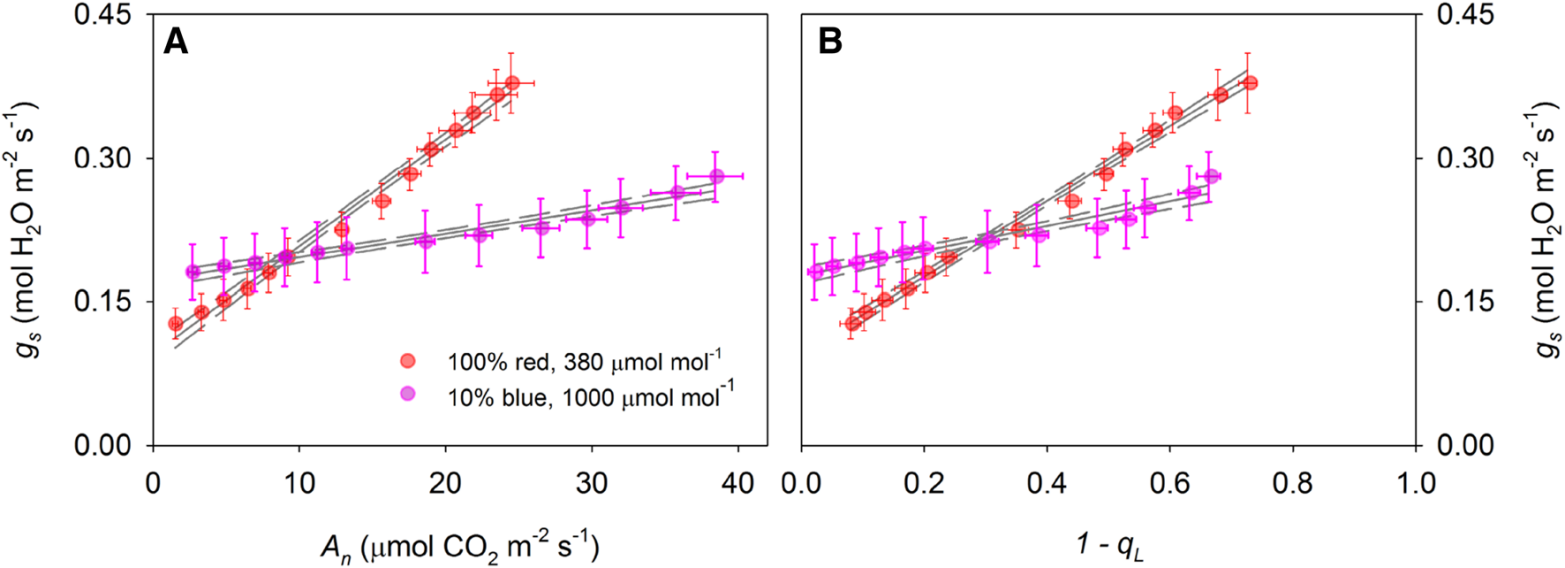
**a** Stomatal conductance (*g*_s_) plotted as a function of net assimilation rate (*A*_*n*_). **b** Stomatal conductance plotted as a function of fluorescence parameter 1 − *q*_L_. Red symbols indicate measurements performed at CO_2_ concentration in the cuvette of 380 µmol mol^−1^, 100% red light, purple symbols indicate measurements at CO_2_ of 1000 µmol mol^−1^, 90% red 10% and blue light. Solid and dashed lines show linear regressions and 95% confidence intervals, respectively. Error bars indicate standard errors (*n* = 6–7 biological replicates)

**Fig. 2:**
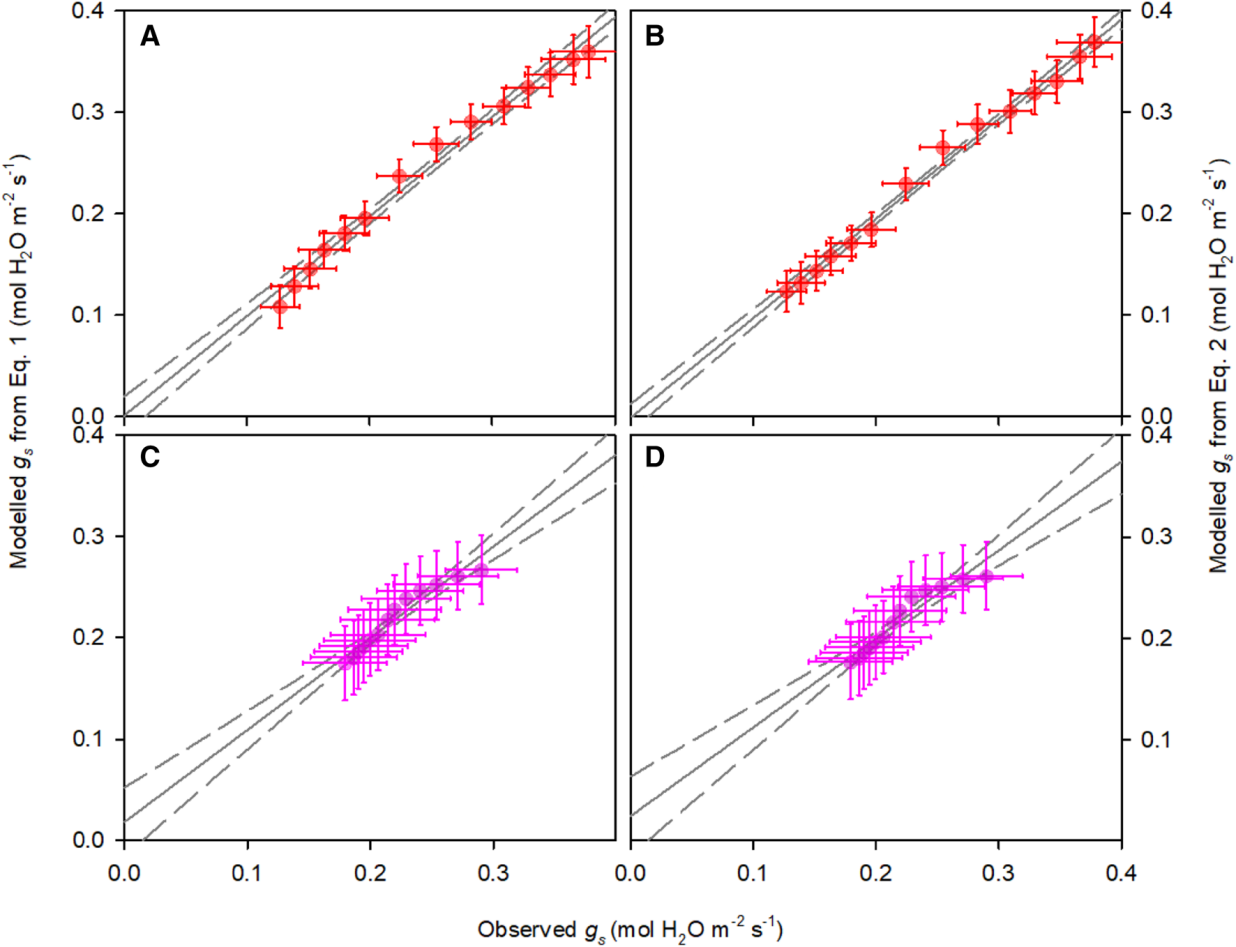
Measured versus modelled stomatal conductance (*g*_s_). Stomatal conductance was modelled for measurements performed at CO_2_ concentration in the cuvette of 380 µmol mol^−1^ and 100% red light with Eq. (1) (Medlyn et al. 2011, panel a) and with the modified model (Eq. 2, panel b) and for measurements using 90% red and 10% blue light and CO_2_ of 1000 µmol mol^−1^ with the Medlyn model (panel c) and with the modified model (panel d). Solid and dashed lines show linear regressions and 95% confidence intervals, respectively. Error bars indicate standard errors (*n* = 6 biological replicates)

**Fig. 3:**
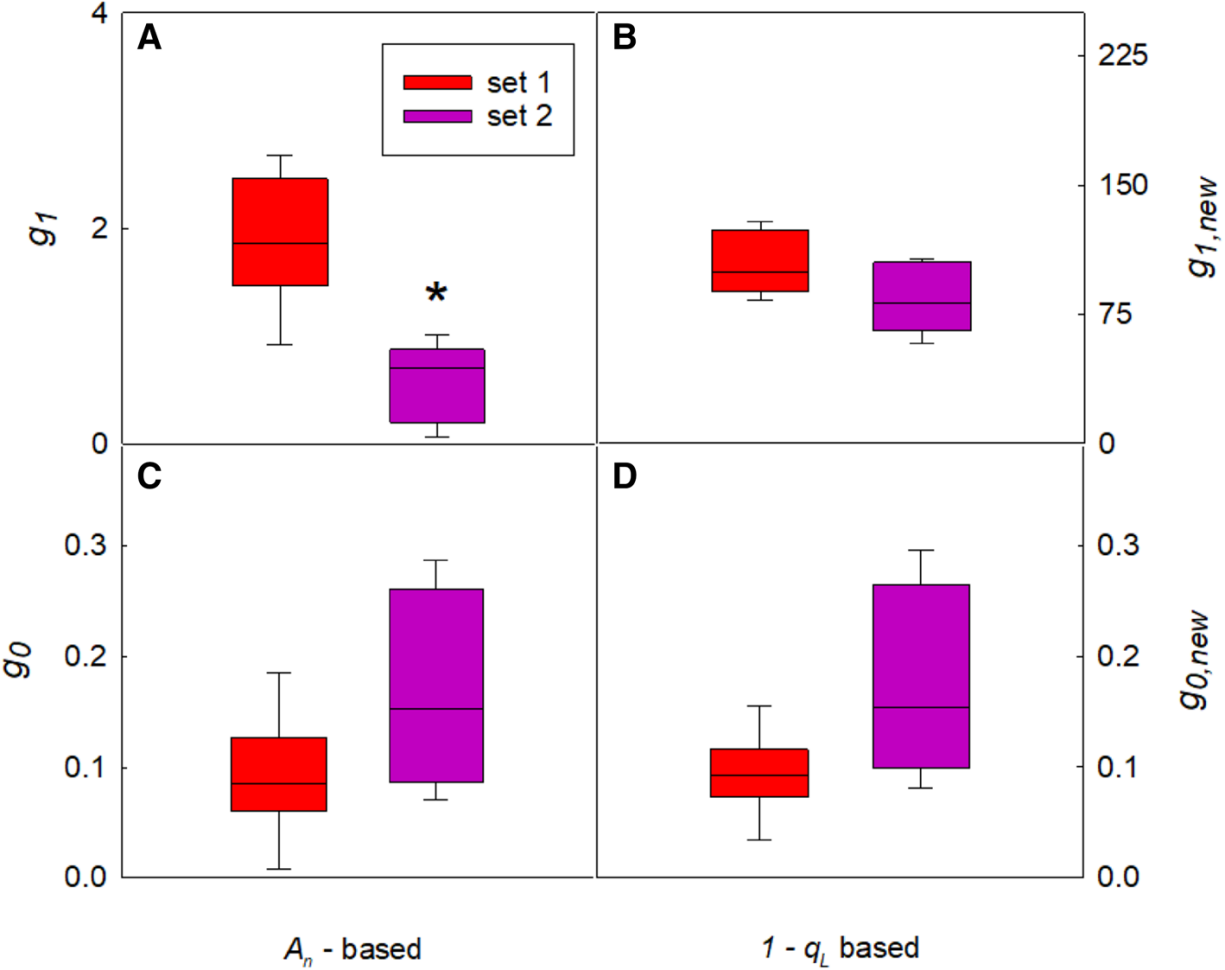
Estimated model parameters (*g*_0_, *g*_0,new_, *g*_1_, *g*_1,new_) for the stomatal conductance model with either *A*_*n*_ (panel **a** and **c**) or 1 − *q*_L_ (panel **b** and **d**) as the estimator for light-induced stomatal movements. Light response curves were measured with either 100% red light and 380 µmol mol^−1^ CO_2_ (set 1) or 90% red and 10% blue light and 1000 µmol mol^−1^ CO_2_ (set 2). Asterisk indicates significant difference between parameter estimate for set 1 versus set 2 (*p* = 0.001, Student’s *t* test, *n* = 6)

The measured light responses of stomatal conductance showed highly significant linear correlations with both *A*_*n*_ and *1* − *q*_L_ (*p* < 0.005, Fig. 1a, b). The slopes of the regressions were significantly different between the two sets of light response curves (Student’s *t* test, *p* < 0.05), as could be expected from the well-known suppression effect of high CO_2_ on stomatal movements. The slope of both stomatal conductance models is essentially a linear multiplication of response factors (i.e. ${A_n}\, \times \,C_{{\text{a}}}^{{ - 1}} \times {\text{VPD}}_{{\text{A}}}^{{ - 0.5}}\;{\text{or}}\;(1 - {q_{\text{L}}})\, \times \,C_{{\text{a}}}^{{ - 1}} \times {\text{VPD}}_{{\text{A}}}^{{ - 0.5}})$. Therefore, the ratio of the slopes of the regressions of *g*_s_ against either *A*_*n*_ or 1 − *q*_L_ should equal ${{\left( {C_{{\text{a}}}^{{ - 1}} \;{\text{set}}1\; \times \;{\text{VPD}}_{A}^{{0.5}} \;{\text{set}}1} \right)} \mathord{\left/ {\vphantom {{\left( {C_{{\text{a}}}^{{ - 1}} \;{\text{set}}1\; \times \;{\text{VPD}}_{A}^{{0.5}} \;{\text{set}}1} \right)} {\left( {C_{{\text{a}}}^{{ - 1}} \;{\text{set}}2\; \times \;{\text{VPD}}_{A}^{{0.5}} \;{\text{set}}2} \right)}}} \right. \kern-\nulldelimiterspace} {\left( {C_{{\text{a}}}^{{ - 1}} \;{\text{set}}2\; \times \;{\text{VPD}}_{A}^{{0.5}} \;{\text{set}}2} \right)}},$ which was calculated to be 2.27. The measured slope ratio between the linear regressions in Fig. 1a (*A*_*n*_ vs. *g*_s_) was 4.67, whereas the regressions in Fig. 1b (1 − *q*_L_ vs. *g*_s_) showed a slope ratio of 3.06, which was considerably closer to the predicted value. This suggests that the relationship between *g*_s_ and 1 − *q*_L_ is more conserved than between *g*_s_ and *A*_*n*_ when measurement conditions are varied. This was also confirmed by fitting the model parameters *g*_0_ and *g*_1_ (Fig. 2a, c) or *g*_0,new_ and *g*_1,new_ (Fig. 2b, d) for each individual light response curve. One light response curve in set 2 did not converge to a reasonable estimate for *g*_1_ in the Medlyn model, and was discarded to avoid confounding the comparison between *A*_*n*_ and 1 − *q*_L_. For the remaining 12 light response curves, variation in stomatal conductance was adequately captured by both models. However, whereas the fitted slope parameter *g*_1_ decreased significantly by 58% for measurements at 1000 µmol mol^−1^ CO_2_ and 10% blue compared to 380 µmol mol^−1^ CO_2_ and 100% red light (1.90 ± 0.25 vs. 0.60 ± 0.15, *p* = 0.001, Fig. 3a), fitted *g*_1,new_ did not vary significantly (103 ± 8 vs. 84 ± 8, *p* = 0.10, Fig. 3b).

### Predicting *q*_L_ with the extended photosynthesis model

**Fig. 4:**
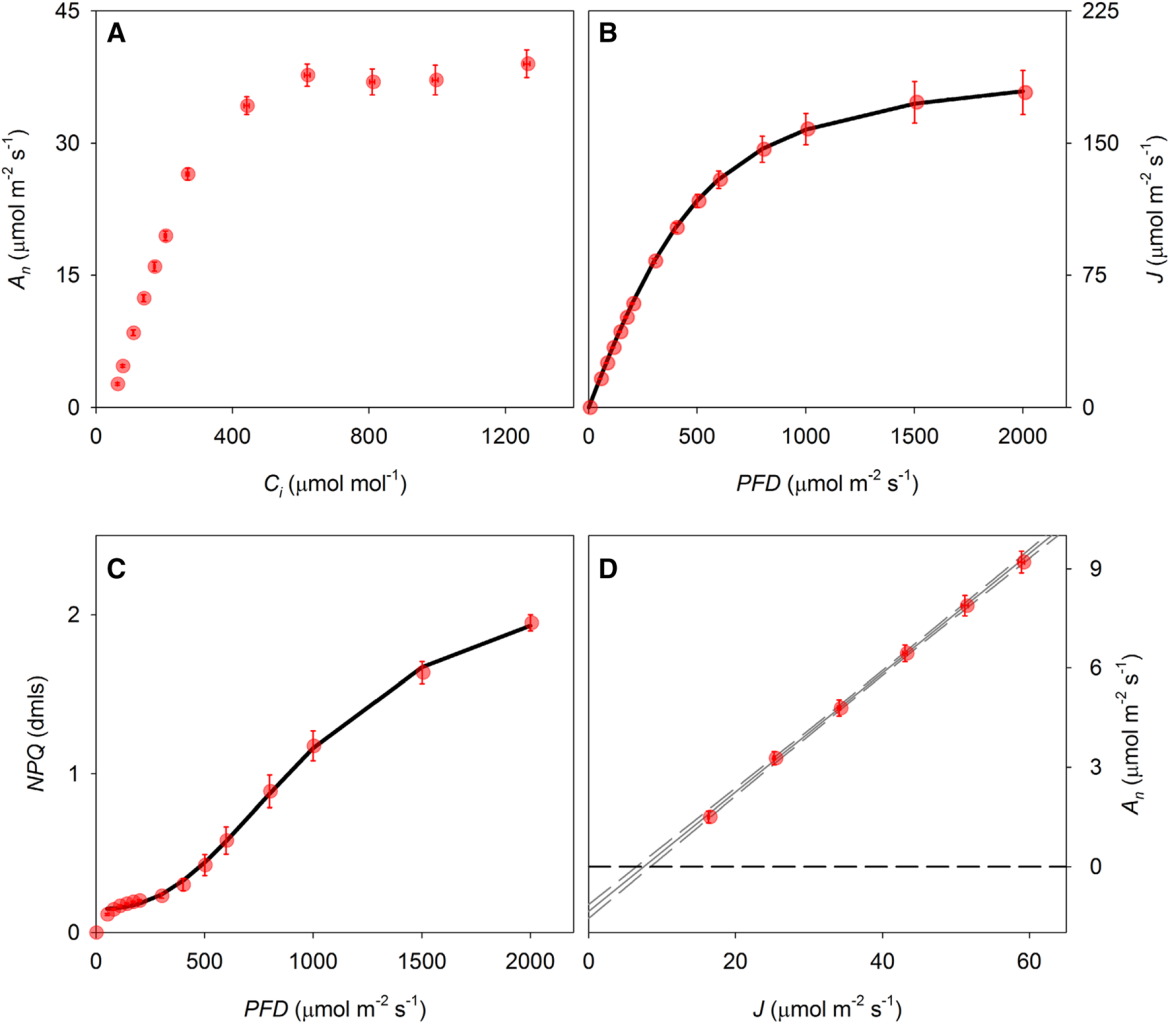
Response curves to derive model parameters for the photosynthesis model. Net assimilation rate *A*_*n*_ plotted as a function of **a** intercellular CO_2_ concentration (*C*_i_), **b** whole-chain electron transport (*J*) and **c** non-photochemical quenching (NPQ) plotted as a function of incident light (PFD) and **d***A*_*n*_ plotted as a function of *J*. Solid lines in **b** and **c** depict model fits (Eqs. 4 and 6). The data in **d** were used to estimate mitochondrial respiration rate not associated with photorespiration (*R*_d_) as the *y* intercept of the linear correlation. Solid and dashed lines in **d** show linear regressions and 95% confidence intervals, respectively. Error bars indicate standard errors (*n* = 6 biological replicates)

**Fig. 5:**
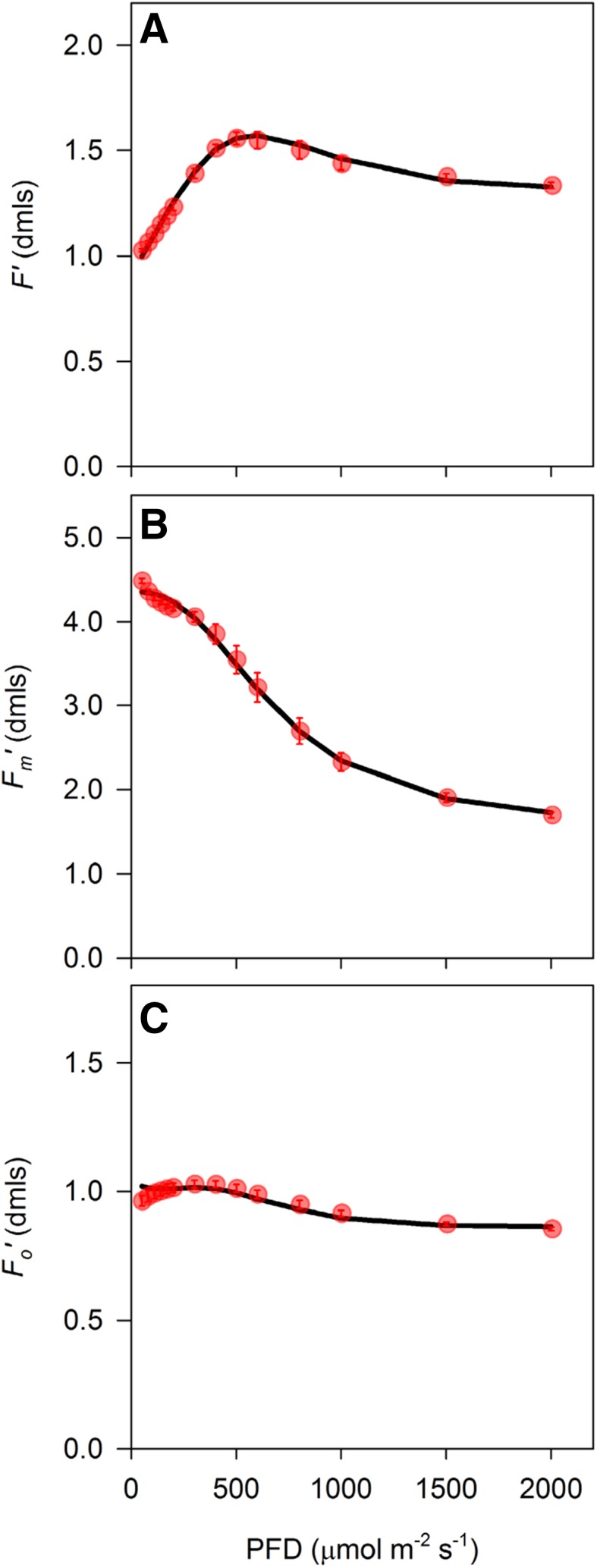
**a** Steady state fluorescence (*F*′), **b** maximal fluorescence under illumination (${F^{\prime}_{\text{m}}}$) and **c** minimal fluorescence under illumination (${F^{\prime}_{\text{o}}}$). Symbols indicate measurements (scaled to corresponding *F*_*m*_ measurement), solid lines show model simulations. Error bars indicate standard errors (*n* = 6 biological replicates)

**Fig. 6:**
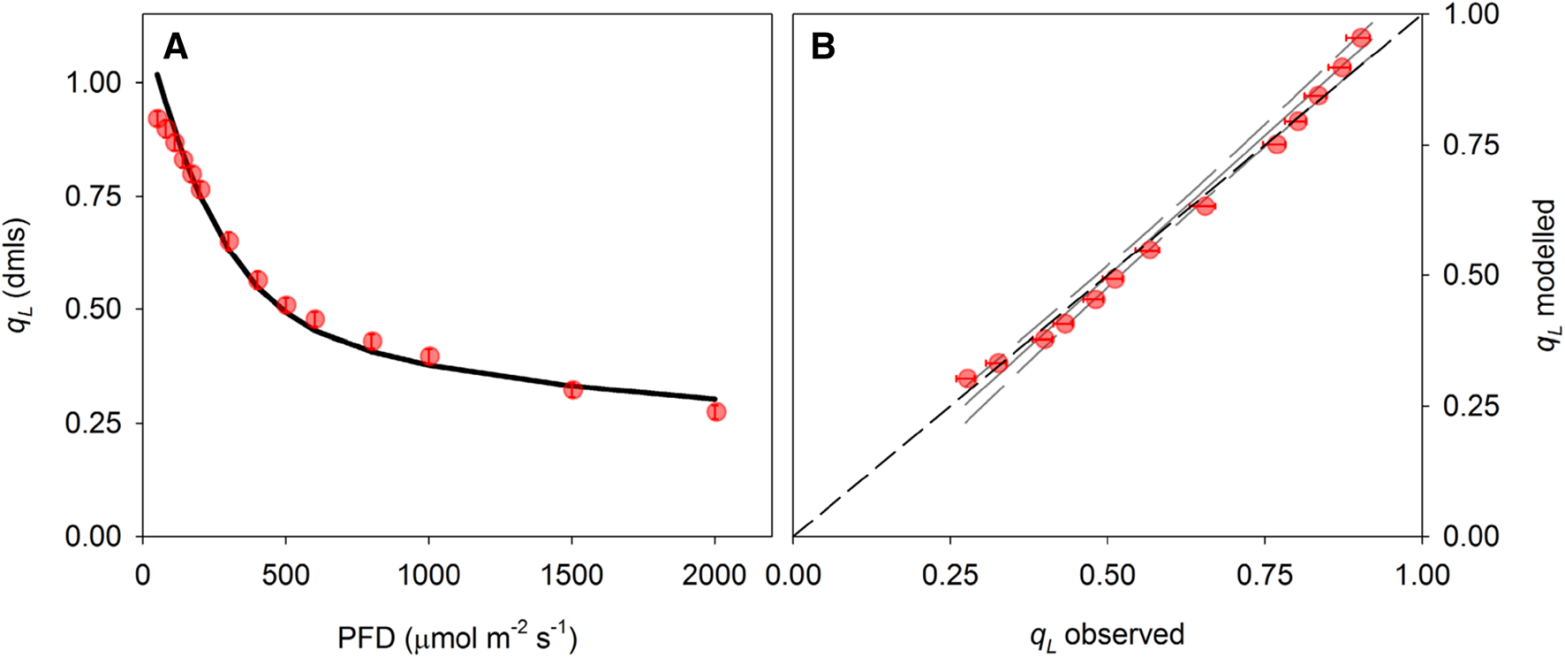
**a** Fluorescence parameter *q*_L_ as a function of light intensity (PFD), symbols indicate measurements, solid line shows model simulations. **b** Correlation between observed and modelled *q*_L_ shown in panel **a**. Solid and dashed lines in **b** depict linear regression (*y* = 1.08*x* − 0.04) and 95% confidence intervals, respectively. Slope and intercept did not deviate significantly from *x* = *y* shown by the black dashed line (*p* > 0.05). Error bars indicate standard errors (*n* = 6 biological replicates)

To facilitate the integration of 1 − *q*_L_ as a predictor of light-induced stomatal movements in higher level models, we extended the widely used FvCB biochemical model for leaf photosynthesis (Farquhar et al. 1980) to allow simulation of *q*_L_. First, leaf biochemical capacity for RuBP carboxylation (${V_{{{\text{c}}_{{\text{max}}}}}}$) and triose phosphate utilization (*V*_TPU_) were estimated based on CO_2_ response curves (Fig. 4a). Light response curves were used to parameterize descriptive equations for whole-chain electron transport rate *J* (Fig. 4b) and non-photochemical quenching NPQ (Fig. 4c) and estimate the rate of mitochondrial respiration in the light (*R*_d_) as the *y* intercept of the initial linear response of *A*_*n*_ to *J* (Fig. 4d). All parameter estimates are shown in Table 1 in “Appendix”. These estimates were then used to simulate fluorescence parameters ${F^{\prime}_{\text{m}}}$, *F*′, and ${F^{\prime}_{\text{o}}}$ (Fig. 5a–c). Simulation of ${F^{\prime}_{\text{o}}}$ showed a slight mismatch compared to the measured values at low light intensity, which is due to the fact that the relationship described in Eq. (11) becomes slightly curvi-linear at low light. However, the overall fit between measured and simulated fluorescence parameters was adequate to accurately reproduce most of the observed variation in *q*_L_ (*R*^2^ = 0.984) and the linear correlation did not differ significantly from *x* = *y* (*p* > 0.05, Fig. 6a, b).

### Coupled model for photosynthesis and stomatal conductance

**Fig. 7:**
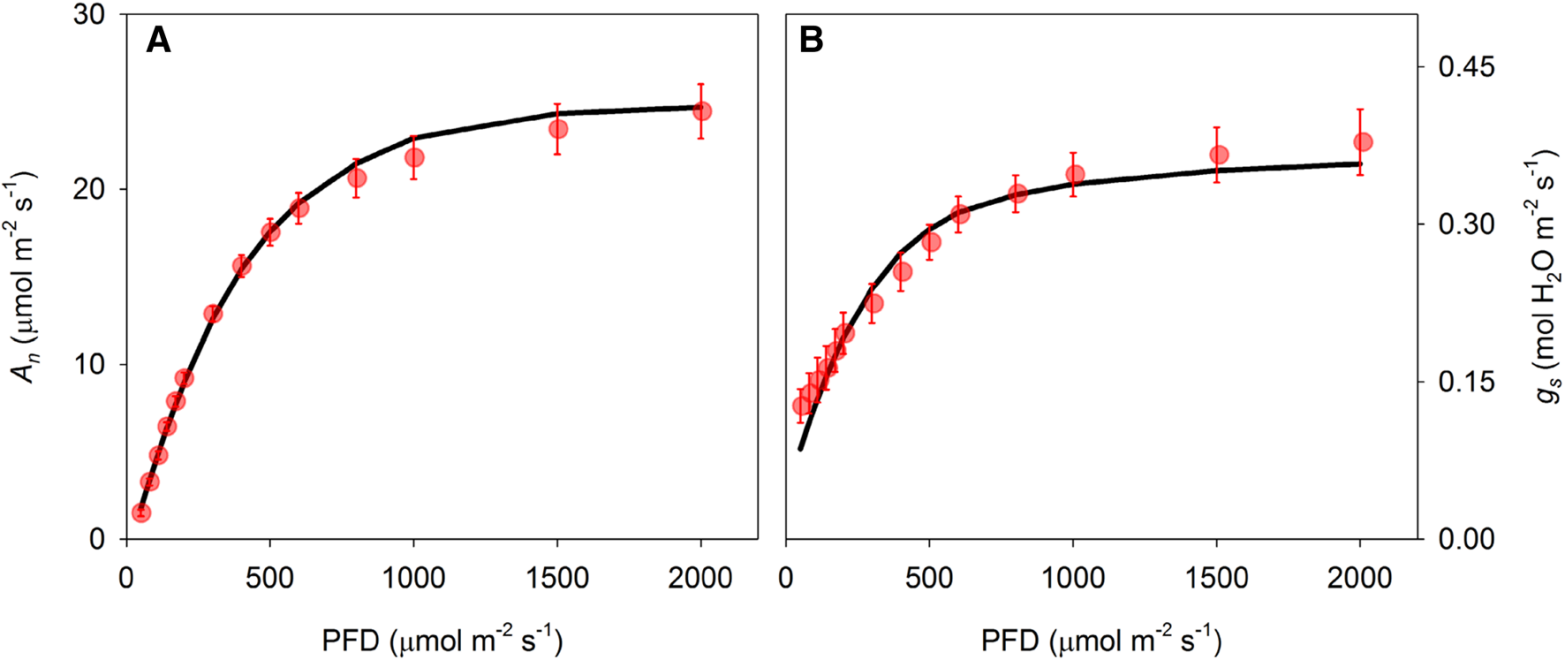
Observed and modelled *A*_*n*_ (**a**) and *g*_s_ (**b**) as a function of light intensity. Model simulations were performed with the coupled model for photosynthesis and stomatal conductance. Error bars indicate standard errors (*n* = 6 biological replicates)

Simulation of *q*_L_ through the extended photosynthesis model shown in Fig. 6 provided a coupling point for the modified stomatal conductance model. The coupled stomatal conductance–photosynthesis model was used to simulate *A*_*n*_ (Fig. 7a) and *g*_s_ (Fig. 7b) as a function of light intensity by iteratively solving differences between the two equations for intercellular CO_2_ concentration *C*_i_ (Eqs. 13 and 14). Both were simulated reasonably accurately across the light response, although a slight mismatch in the curvature of *g*_s_ was observed (Fig. 7b).

**Fig. 8:**
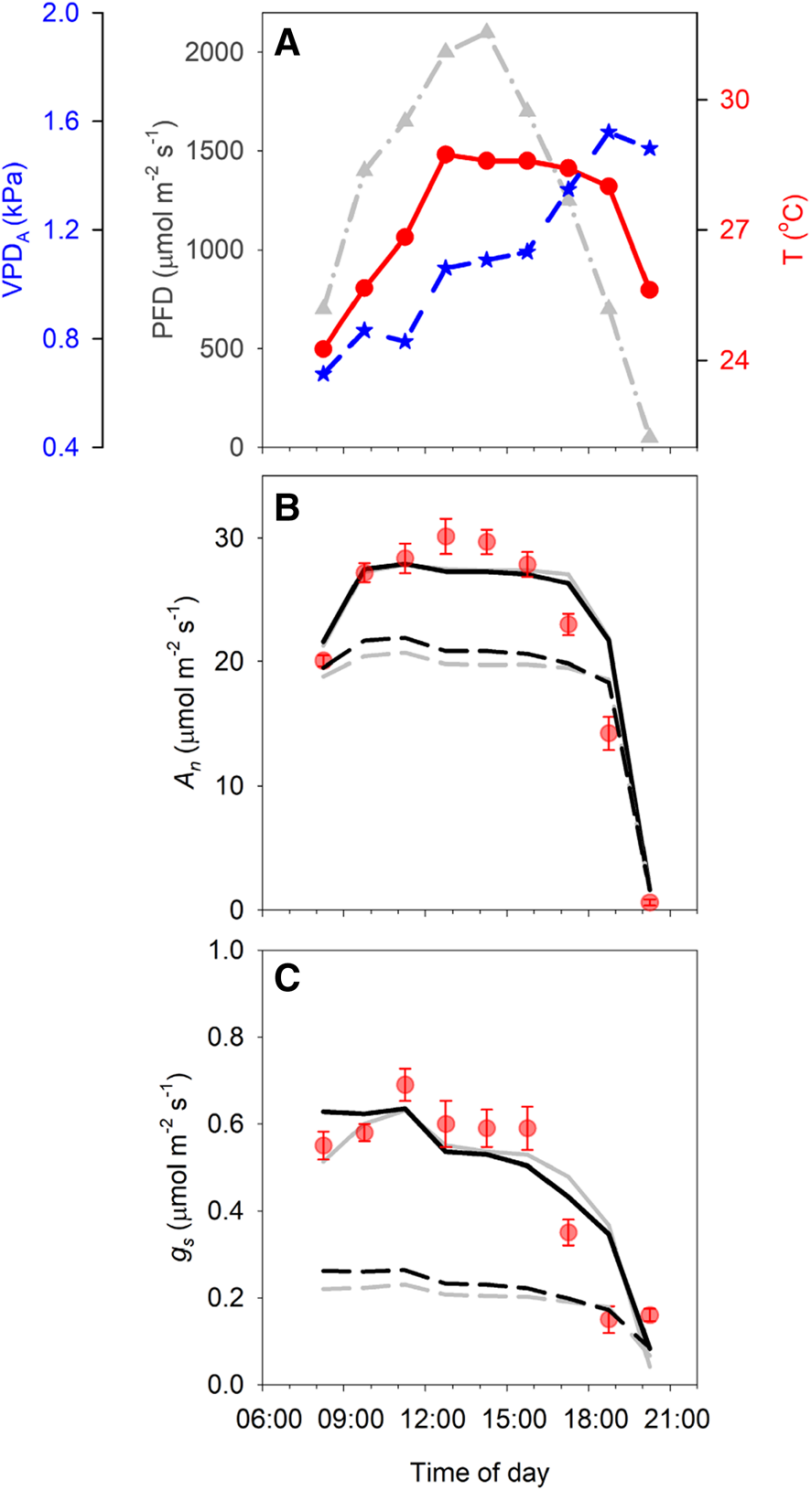
**a** Diurnal measurements of light intensity (PFD), air temperature (T) and air vapour pressure deficit (VPD_A_) during July 21, 2015 in Urbana, Illinois, USA. **b** Observed (symbols) and modelled (lines) net assimilation rate (*A*_*n*_) at 90 min intervals. Simulations were performed with the coupled photosynthesis-stomatal conductance model, using the weather data in **a** as input. Lines show model predictions using either the Medlyn (grey lines) or the modified stomatal conductance model (black lines), with parameter estimates from controlled conditions (dotted lines) or re-calibrated on field-grown plants (solid lines) (**c**) Observed (symbols) and modelled (lines) stomatal conductance (*g*_s_). Line legend as explained for **b**. Error bars indicate standard errors (*n* = 7–8 biological replicates)

As an independent verification, diurnal gas exchange measurements on field-grown tobacco were used to further test the performance of the coupled model. Measurements were performed on a well-watered tobacco crop on a hot, clear day in mid-summer (Fig. 8a). The first measurement point was taken at 08.15, when light intensity had already reached 700 µmol m^−2^ s^−1^ and *A*_*n*_ and *g*_s_ were already quite high (averaging 20.0 µmol m^−2^ s^−1^ and 0.55 mol m^−2^ s^−1^, respectively; Fig. 8b and c). Subsequent measurements showed a slight increase in *g*_s_ towards 11.15 followed by a gradual decline throughout the afternoon. *A*_*n*_ also increased towards mid-day, reaching maximum values somewhat later than *g*_s_, at 12:45 and 14:15, followed by a gradual decline throughout the remainder of the photoperiod.

To simulate these observations, we used both stomatal conductance models with either the parameter estimation from plants grown under controlled conditions or re-calibrated on the field-grown plants, while the photosynthesis model was parameterized on the field-grown plants at all simulations. Using the parameter estimates from controlled conditions for either the Medlyn or modified stomatal conductance model resulted in severe underestimation of stomatal conductance and net assimilation rate (Fig. 8b, c) as could be expected based on known differences in stomatal acclimation between controlled versus field conditions. A better match between modelled and observed data was obtained by re-calibration of the stomatal conductance model by minimizing the residuals between modelled and observed *g*_s_. Model predictions with re-calibrated parameters showed a reasonable match with observed *A*_*n*_ and *g*_s_ for the majority of the time-points except for late in the photoperiod (17:15 and 18:45), where *A*_*n*_ and *g*_s_ were lower than predicted by the model. The minimized residuals were marginally smaller for the modified model compared to the Medlyn model, (0.115 vs. 0.123). In addition, the residuals across a wide range of parameter values remained considerably lower in the modified stomatal conductance model, compared to the Medlyn model (0.115–0.162 vs. 0.123–0.210 for parameter values shown in Fig. S2).

## Discussion

### Modelling light-induced stomatal movements

Models for stomatal conductance are important components of canopy, ecosystem, land surface and even earth system models in predicting future climate and biosphere productivity. Here we have shown that the widely used empirical BWB model for stomatal conductance (version by Medlyn et al. 2011) can be changed to incorporate the putative causal relationship between PQ redox state and light-induced stomatal movements (Busch 2014; Głowacka et al. 2018) yet kept simple enough to facilitate easy integration in models of greater scale. The modification was shown to lead to more conserved estimates for the slope parameter *g*_1,new_ across different measurement conditions, which should help to increase confidence in predictions under future climates. The modifications to the model arguably represent a more mechanistic basis for stomatal responses to light, compared to the Medlyn model, although it is still very empirical and simplistic. Whereas more mechanistic models can typically be expected to do a better job in generating new insights and predicting *g*_s_ outside the validated range (Buckley 2017), they have a tendency to become too complex or include difficult to estimate parameters, which can make inclusion in levels of greater scales tricky. Therefore, there is still a need to refine empirical models such as presented here.

Although tobacco guard cells are known to respond only very weakly to the addition of blue light (Marten et al. 2008), it is possible that the slope parameters *g*_1_ and *g*_1,new_ may not strictly represent only the quantitative stomatal ‘red’ light response for the second set of response curves. Further testing in different species will need to be done to verify this. Interestingly, the over-excitation of photosystem II compared to photosystem I by blue light may directly promote a more reduced PQ redox state, which is hypothesized to lead to stomatal opening (Busch 2014; Głowacka et al. 2018). If so, the putative causal relationship between 1 − *q*_L_ and *g*_s_ implies that the stronger response of stomatal conductance to blue light may also arise via the ‘red light’ response, i.e. without the phototropin signalling cascade, although this effect would be more apparent at higher ratios between red and blue light than 9:1 used here and would also depend on parallel effects on induction of NPQ.

### Estimation of NPQ and *q*_L_

To couple the new model of *g*_*s*_ based on 1 − *q*_L_ with other models, requires accurate prediction of *q*_L_. We have presented a simple extension to the widely used FvCB model for photosynthesis (Farquhar et al. 1980), which is easy to parameterize and can be used to predict *q*_L_ reasonably well across a range of light intensities (Fig. 6). To circumvent the need for dark measurements for ${F^{\prime}_{\text{o}}}$, we simulated non-photochemical quenching and photo-inactivation effects on *F*_o_. For non-photochemical quenching effects we used the formulation for ${F^{\prime}_{\text{o}}}$ by Oxborough and Baker (1997), which simulates the decrease in ${F^{\prime}_{\text{o}}}$ based on the decrease in ${F^{\prime}_{\text{m}}}$ relative to *F*_m_. The fluorescence increase due to photo-inactivation was simulated by an empirical relationship with the estimated energy flux through non-photochemical dissipation pathways (fluorescence, as well as regulated and constitutive thermal dissipation), which has been shown to be linearly correlated with the rate coefficient of photo-inactivation of PSII reaction centres (Hendrickson et al. 2005). This relationship was calibrated on the differences between ${F^{\prime}_{{\text{oNPQ}}}}$ and measured ${F^{\prime}_{\text{o}}}$ (by turning off actinic light and application of weak far-red illumination) under controlled conditions, which confirmed a strong linear relationship, except for very low PFD where the relationship tended to be slightly curvi-linear.

The model simulations of *q*_L_ further depend on accurate estimation of NPQ. We chose to use an empirical sigmoidal Hill function, which was sufficient to demonstrate the use of 1 − *q*_L_ as a predictor of light-induced stomatal movements, but carries limited biological meaning. Additionally, treatment of *NPQ* as an independent parameter does not take account of the intimate connection between photosynthesis and thermal dissipation of absorbed light energy in the photosynthetic antenna complexes. The presented approach may therefore be improved by linking the description of NPQ by Eqs. (6a, 6b) to the parameters describing photosynthetic capacity such as $V_{\text{cmax}}$ and *J*_max_, or using altogether more mechanistic models for simulation of photosynthesis and non-photochemical quenching (e.g. Zaks et al. 2012; Morales et al. 2018). Interestingly, the estimation of NPQ at larger scales has gained a lot of interest recently due to development of gross primary productivity (GPP) proxies based on ground-based or remotely sensed measurements of solar induced fluorescence (SIF, reviewed by Porcar-Castell et al. (2014) and several others). Here, the interaction between steady state photosynthesis and the passive emission of chlorophyll fluorescence provides an optical signal which can be used to estimate GPP. However, since steady state fluorescence is the product of absorbed light and the quantum yield of fluorescence, both photochemical and non-photochemical quenching can affect the SIF signal. Hence, additional modelling or parallel proxies for NPQ are required in order to use SIF signals as a proxy for GPP. One often-used proxy for NPQ is the photochemical reflectance index (PRI, Gamon et al. 1992), which is based on the broadband scattering change at 531 nm associated with pigment conversions in the xanthophyll cycle and a conformational change in the PSII antenna, which accompanies energy-dependent quenching (Bilger and Bjorkman 1994; Johnson et al. 2009). Short-term (diurnal) variations in the PRI signal can be successfully used to provide a proxy for canopy or ecosystem light use efficiency (Gamon et al. 1997; Hilker et al. 2011). Our demonstration that 1 − *q*_L_ can be used as a proxy for light-induced stomatal movements suggests that in addition to light use efficiency, optical proxies such as PRI may also turn out to be useful in constraining ecosystem water vapour exchange estimates based on stomatal conductance in terrestrial biosphere models.

### More robust estimation of *g*_s_ across different conditions

We have demonstrated that using the fluorescence parameter 1 − *q*_L_ instead of *A*_*n*_ makes the slope parameter in the stomatal conductance model (*g*_1_ and *g*_1,new_) more robust against differing measurement conditions (Fig. 3). In addition, residuals of the modified stomatal conductance model were consistently lower than for the Medlyn model across a wide range of parameter values (Fig. S2). This is of great value to increase confidence in predictions of vegetation responses to future climate conditions. Slope and intercept parameters of BWB model (Ball et al. 1986) and the derivation by Medlyn et al. (2011) have been reported to vary substantially between species, and species-specific parameterization greatly improved model predictions of *A*_*n*_ and *g*_s_ (Wolz et al. 2017). If the light response of stomatal opening is indeed mechanistically connected to the PQ redox state, the modified model may also provide a more generic parameterization across species, but more measurements on different species will be needed to assess this. However, although the species-specific differences between slope and intercept parameters as shown by Wolz et al. (2017) may have been aggravated by using *A*_*n*_ as an estimator of the stomatal light response, it is very likely that considerable species-specific parameterization will remain necessary in the modified model. For instance, whereas the blue-light response of guard cells is relatively weak in tobacco, which allowed lumping it in with the quantitative response in a single slope parameter *g*_1,new_, this may possibly require more explicit parameterization in species with a stronger response to blue light. The level of NPQ is also known to vary between species (Demmig-Adams 1998), within species (Jung and Niyogi 2009; Kasajima et al. 2011; Ortiz et al. 2017) and with leaf age and plant development stage (Bielczynski et al. 2017) and the same is true for photosynthetic capacity and leaf morphology. This is also evident from the model simulations of field-grown tobacco, where better fits could be obtained with substantially increased slope parameters (*g*_1_, *g*_1,new_) and decreased intercepts (*g*_0_, *g*_0,new_; Fig. 8 and Fig. S2). Different parameter values are to be expected based on known differences in stomatal acclimation between controlled and field conditions (Matthews et al. 2018). Late in the photoperiod, both models overestimated *A*_*n*_ and *g*_s_. This may require more detail in the simulation of stomatal responses to vapour pressure deficit, leaf water status or long-term diurnal stomatal movements. For example, inclusion of a diurnal sinusoidal pattern in the BWB stomatal conductance model greatly improved prediction accuracy (Matthews et al. 2018). The physiological basis for these diurnal stomatal movements is not entirely clear, but circadian regulation (Hassidim et al. 2017) and interactions with sugar and ethylene signals (Kelly et al. 2013; Haydon et al. 2017) are well-known to have an impact on stomatal conductance. It is also clear that 1 − *q*_L_ will be subject to much faster changes than stomatal responses which suggests that the slower stomatal responses may reflect a time-averaged redox signal initiated at the chloroplastic PQ pool. Interestingly, the use of 1 − *q*_L_ in the stomatal conductance model would also allow the kinetic behaviour of NPQ to impact stomatal dynamic properties, similar to our findings for steady state values (Głowacka et al. 2018). Namely, build-up of sustained NPQ throughout the photoperiod would directly dampen the signal for stomata to open in response to light. Further work is needed to test the relationship between PQ redox state and red light-induced stomatal movements. The presented model equations provide a structured framework to generate and verify hypotheses based on this putative relationship.

### Electronic supplementary material

Below is the link to the electronic supplementary material.

Supplementary material 1 (DOCX 113 KB)

## Appendix

**Table 1:** Model parameter estimates under controlled and field conditions

Parameter name	Description	Unit	Greenhouse (value at 25 °C)	Field (value at 25 °C)	Source
*g*_0_	Intercept parameter in Medlyn stomatal conductance model	mol H_2_O m^−2^ s^−1^	0.091	0.027	Equation (1) fit on light response curves (controlled conditions) or combined photosynthesis-stomatal conductance model fit on diurnal data (field)
*g*_1_	Slope parameter in Medlyn stomatal conductance model	Dimensionless	1.90	6.46	Equation (1) fit on light response curves (controlled conditions) or combined photosynthesis-stomatal conductance model fit on diurnal data (field)
*g*_0,new_	Intercept parameter in modified stomatal conductance model	mol H_2_O m^−2^ s^−1^	0.093	0	Equation 2 fit on light response curves (controlled conditions) or combined photosynthesis-stomatal conductance model fit on diurnal data (field)
*g*_1,new_	Slope parameter in modified stomatal conductance model	Dimensionless	104	322	Equation (2) fit on light response curves (controlled conditions) or combined photosynthesis-stomatal conductance model fit on diurnal data (field)
$V_{\text{cmax}}$	Maximal rate of RuBP carboxylation	µmol m^−2^ s^−1^	121	115.2	Fitted on CO_2_ response curves
*V*_TPU_	Maximal rate of triose phosphate utilization	µmol m^−2^ s^−1^	11.5	14.1	Fitted on CO_2_ response curves
*g*_m_	Mesophyll conductance to CO_2_ transfer	mol CO_2_ m^−2^ s^−1^ bar^−1^	0.60	0.60	Derived from carbon isotope measurements
*R*_d_	Mitochondrial respiration not associated with photorespiration, under illuminated conditions	µmol m^−2^ s^−1^	1.35	1.16	Estimated as *y* intercept of linear correlation between *A*_*n*_ versus *J* under light limited range
*J*_max_	Maximal rate of whole-chain electron transport (*J*)	µmol m^−2^ s^−1^	205	200	Estimated from fitting non-rectangular hyperbole to chlorophyll fluorescence measurements during light response curves
*α*	Initial slope non-rectangular hyperbolic fit of *J* response to light intensity	Electrons/photons	0.79	0.72	Estimated from fitting non-rectangular hyperbole to chlorophyll fluorescence measurements during light response curves
*θ*	Shape factor non-rectangular hyperbolic fit of *J* response to light intensity	Dimensionless	0.74	0.75	Estimated from fitting non-rectangular hyperbole to chlorophyll fluorescence measurements during light response curves
*f*_PSII_	Proportion of absorbed light partitioned to PSII	Dimensionless	0.50	0.50	Not estimated here
NPQ_max_	Asymptote value sigmoidal fit of non-photochemical quenching (NPQ) response to light intensity	Dimensionless	2.24	2.81	Estimated from fitting sigmoidal Hill function to chlorophyll fluorescence measurements during light response curves
NPQ_0_	Basal NPQ value	Dimensionless	0.15	0.42	Estimated from fitting sigmoidal Hill function to chlorophyll fluorescence measurements during light response curves
*K*_NPQ_	Light intensity at half amplitude of NPQ	Dimensionless	1042	1672	Estimated from fitting sigmoidal Hill function to chlorophyll fluorescence measurements during light response curves
*n*_NPQ_	Apparent Hill coefficient for NPQ response to light	Dimensionless	2.52	2.28	Estimated from fitting sigmoidal Hill function to chlorophyll fluorescence measurements during light response curves
*m*	Slope parameter to estimate effect of reaction centre inactivation on minimal fluorescence (${F^{\prime}_{\text{o}}}$)	Dimensionless	2.34 × 10^−4^	2.34 × 10^−4^	Estimated from fitting Eq. (11) on chlorophyll fluorescence measurements during light response curves
*n*	Intercept parameter to estimate effect of reaction centre inactivation on ${F^{\prime}_{\text{o}}}$	Dimensionless	0.038	0.038	Estimated from fitting Eq. (11) on chlorophyll fluorescence measurements during light response curves
