# Supplementary material for: Predicting light-induced stomatal movements based on the redox state of plastoquinone: theory and validation
Source: Photosynth Res. 2019 Mar 19;141(1):83–97. doi: 10.1007/s11120-019-00632-x (PMC6612513; doi:10.1007/s11120-019-00632-x)
Supplement: Supplementary file 1 — Supplementary material 1 (DOCX 113 KB) [file 11120_2019_632_MOESM1_ESM.docx]

**Fig. S1** Schematic of measurements and model parameterization.

**Fig. S2** Residuals for modelled compared to observed stomatal conductance (*g_s_*) under field conditions shown in Fig. 8A. (A) Residuals for predictions with the Medlyn stomatal conductance model coupled to the photosynthesis model at a wide range of values for *g_0_* and *g_1_* (depicted as percentage of the values for controlled-conditions grown plants). (B) Same as (A) but for the modified model. Legend shows color-coding for residuals.
